# Unveiling a new oceanic anoxic event at the Norian/Rhaetian boundary (Late Triassic)

**DOI:** 10.1038/s41598-024-66343-z

**Published:** 2024-07-06

**Authors:** Manuel Rigo, Xin Jin, Linda Godfrey, Miriam E. Katz, Honami Sato, Yuki Tomimatsu, Mariachiara Zaffani, Matteo Maron, Sara Satolli, Giuseppe Concheri, Alessandra Cardinali, Qiangwang Wu, Yixing Du, Jerry Zhen Xiao Lei, Connor S. van Wieren, Lydia S. Tackett, Hamish Campbell, Angela Bertinelli, Tetsuji Onoue

**Affiliations:** 1 Department of Geosciences, University of Padova, Via G. Gradenigo 6, 35131 Padova, Italy; 2 IGG-CNR (Istituto Di Geoscienze E Georisorse), Padova, Firenze Italy; 3 State Key Laboratory of Oil and Gas Reservoir Geology and Exploitation and Key Laboratory of Deep-Time Geography and Environment Reconstruction and Applications of Ministry of Natural Resources, Chengdu University of Technology, Chengdu, 610059 China; 4 Department Earth and Planetary Sciences Rutgers, The State University of New Jersey, Piscataway, NJ 08854-8066 USA; 5 Geosciences Dept, Union College, Schenectady, NY 12308 USA; 6 Department of Earth and Planetary Sciences, Kyushu University, Fukuoka, 819-0395 Japan; 7 Department of Engineering and Geology, University “G. d’Annunzio” of Chieti-Pescara, Via Dei Vestini 31, 66100 Chieti, Italy; 8 Department of Agronomy Food Natural Resources Animals and Environment (DAFNAE), University of Padova, Viale Dell’Università, 16, 35020 Legnaro, Italy; 9 School of Earth and Ocean Sciences, University of Victoria, 3800 Finnerty Road, Bob Wright Centre A405. Victoria, Victoria, British Columbia V8P 5C2 Canada; 10 Department of Geological Sciences, Geological Sciences Bldg, University of Missouri, 101, 400 S 6Th St, Columbia, MO 65201 USA; 11 GNS Science, 1 Fairway Drive, 5010 Lower Hutt, Wellington New Zealand; 12 Departmento of Physics and Geology, University of Perugia, Via A. Pascoli, 06123 Perugia, Italy; 13 Department of Earth System Science, Fukuoka University, Fukuoka, 814-0180 Japan

**Keywords:** Palaeoclimate, Palaeontology, Stratigraphy

## Abstract

The latest Triassic was characterised by protracted biotic extinctions concluding in the End-Triassic Extinction (~ 200 Ma) and a global carbon cycle perturbation. The onset of declining diversity is closely related to reducing conditions that spread globally from upper Sevatian (uppermost Norian) to across the Norian-Rhaetian boundary, likely triggered by unusually high volcanic activity. We correlate significant organic carbon cycle perturbations to an increase of CO_2_ in the ocean–atmosphere system, likely outgassed by the Angayucham igneous province, the onset of which is indicated by the initiation of a rapid decline in ^87^Sr/^86^Sr and ^188^Os/^187^Os seawater values. A possible causal mechanism involves elevated CO_2_ levels causing global warming and accelerating chemical weathering, which increased nutrient discharge to the oceans and greatly increased biological productivity. Higher export production and oxidation of organic matter led to a global O_2_ decrease in marine water across the Norian/Rhaetian boundary (NRB). Biotic consequences of dysoxia/anoxia include worldwide extinctions in some fossil groups, such as bivalves, ammonoids, conodonts, radiolarians.

## Introduction

Dramatic middle and late Mesozoic climate events are often associated with significant spread of reducing conditions and linked to increases in extinction rates^1,2^. These discrete episodes are characterised by intense perturbations in the global carbon cycle that typically record low O_2_ conditions and widespread changes in ocean chemistry, called Oceanic Anoxic Events (OAEs)^2–5^. The OAEs are conventionally described as being characterized by black and dark shales with fine laminations and absence of bioturbation, increased sedimentary organic matter and presence of pyrite crystals that attest to low-oxygen conditions^2^. However, the black shale record is sometimes limited in some OAEs or even absent, as occurred for the Paleocene-Eocene Thermal Maximum (PETM)^5,6^. In addition to these sedimentological markers, concentrations and isotopes of several chemical species (e.g., Mo, V, U) are largely applied as redox indicators to identify the OAEs^1,5^. Most OAEs also are characterized by an initial negative δ^13^C excursion (CIE) due to input of light carbon (^12^C) into the ocean–atmosphere system, followed by a positive CIE due to burial of excess ^12^C in organic-rich sediments^1,5,7^. Notably, the position of the CIEs related to OAEs can vary locally when compared to the deposition of the dark to black shales, indicating that the oceanic oxygen depletion events are diachronous across sedimentary basins ^1^. Furthermore, OAEs are generally related to sea water warming indicated by low δ^18^O values, which is however commonly interrupted by cooling phases likely caused by the increase of organic matter burial and *p*CO_2_ decrease^1,5^.

Phanerozoic OAEs are defined by intervals of widespread oxygen depletion in the ocean, recognized by sedimentological and/or geochemical records, yet they are significantly dissimilar from each other^5^. Jurassic and Cretaceous OAEs are particularly well-studied, such as the early Toarcian (*Posidonienschiefer* event, T‐OAE, ~ 183 Ma), early Aptian (Selli event, OAE 1a, ~ 120 Ma), early Albian (Paquier event, OAE 1b, ~ 111 Ma) and Cenomanian–Turonian (Bonarelli event, OAE 2, ~ 94 Ma) events^1,8^. Potential forcing mechanisms for OAEs include massive releases of methane (CH_4_) or volcanogenic carbon dioxide (CO_2_) outgassing into the atmosphere during the emplacement of Large Igneous Provinces (LIPs) as continental flood basalts or oceanic plateaus^1,9^. For example, OAE 1a correlates to the Ontong Java Plateau, OAE 2 is associated with the Caribbean-Colombian Plateau^1^ and the early Toarcian OAE is linked to the Karoo–Ferrar subaerial flood basalt extrusions^10^. Similar scenarios occurred during the late Sevatian (late Norian, Late Triassic) and across the Norian-Rhaetian boundary (NRB), during which significant biotic extinctions of important terrestrial and marine taxa occurred^11^. Notably, cosmopolitan bivalves (e.g., monotids, halobiids) are one of the major fossil groups that suffered a global and severe extinction, starting from the Sevatian to the lowermost Rhaetian^11–13^, while ammonoids suffered the largest decline among invertebrates, with a reduction in genera by more than an order of magnitude^14,15^. Other faunal extinctions and diversity loss also are documented for radiolarians and conodonts^16–18^, marine vertebrates (e.g., actinopterygian fishes) and reptiles (particularly among ichthyosaurs)^19^. In addition, reef communities declined significantly during the Late Triassic^20^. This interval of strong faunal extinctions at the NRB is associated with large carbon isotope excursions recorded on land^21,22^ and in marine environments^11^, which are linked to oscillations of biological pump efficiency and/or ocean stratification^11^.

**Figure 1:**
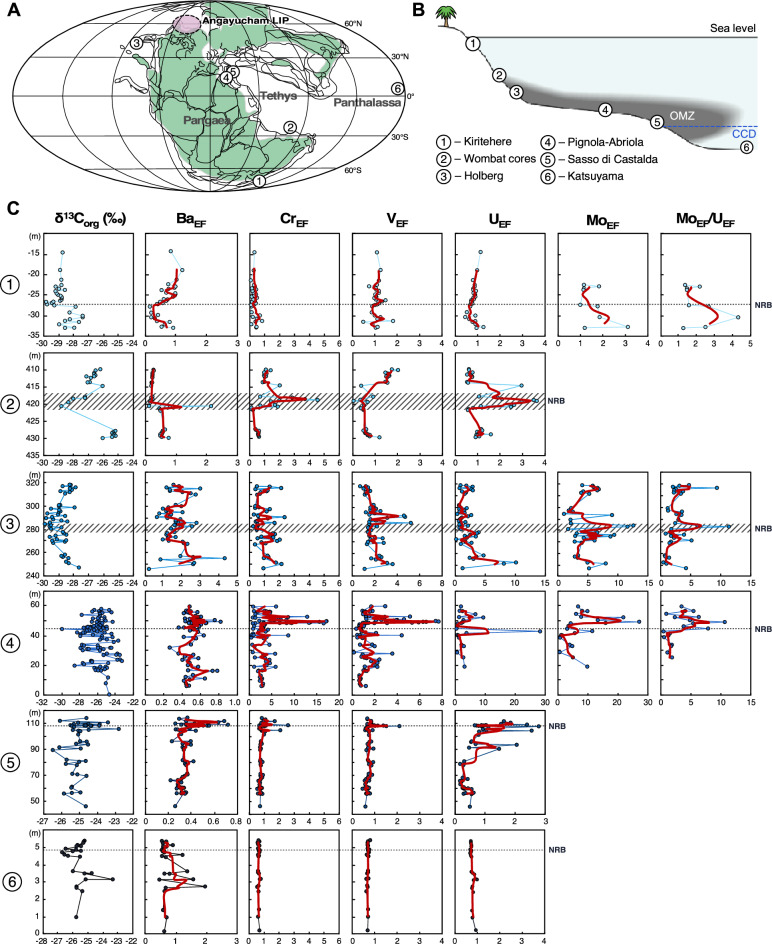
δ^13^C_org_, Ba, and redox-sensitive elements (Mn, Cr, V, U, and Mo) show enrichments in the study sections. The stratigraphic position of the NRB is discussed in Supplementary Information. Blue dots represent raw data, and red lines show moving average on three samples.

We investigated and compared redox conditions recorded in several NRB sections deposited in different sedimentary environments (from shallow to deep water) on opposite sides of Pangea, spanning both hemispheres and across latitudes (Fig. 1). The Pignola-Abriola and Sasso di Castalda sections were deposited in the deep pelagic Lagonegro Basin (Italy), which was part of the western Tethys^23,24^. The Lagonegro Basin records the change from carbonate to biosiliceous sedimentation below the carbonate compensation depth (CCD) across the NRB^25–27^. Both these sections were previously investigated for bio- and chemostratigraphy^11,23–25,27–34^, while magnetostratigraphy was performed only in Pignola-Abriola section^35^. The southern Tethys Wombat Basin (NW Australia) was a neritic continental shelf environment located in the southern Tethys on the margin of Pangea, and was recently studied for biostratigraphy^36^ and chemostratigraphy^11^. The Kiritehere section (New Zealand) was deposited from mid-shelf to deeper water basin on the southernmost margin of Gondwana, close to the South Pole, and it has been recently investigated for organic carbon^37^. The Holberg section (British Columbia, Canada) is illustrated here for the first time and it represents a shallow to moderate depth depositional environment off an oceanic plateau in eastern Panthalassa, and it has been studied for organic carbon stable isotopes (δ^13^C_org_) and chemostratigraphy (this study; Supplementary material). The Katsuyama section (Japan) was deposited below the CCD in the north Equatorial belt of the Panthalassa Ocean has been studied for bio- and chemostratigraphy and δ^13^C_org_ (this study; Supplementary material). All these sections spanned the uppermost Sevatian to lower Rhaetian, which is usually characterized by laminated layers and dark to black shales, except for the Katsuyama section that consists of red and dark red shales and cherts(^11^ and Supplementary material). Notably, the Kiritehere section mostly consists of dark volcanoclastic rocks without any particularly darker portions of the succession. The NRB is identified with the first occurrence of the conodont *Misikella posthernsteini* or (when necessary) is approximated by the negative δ^13^C_org_ peak just below the base of the Rhaetian^11,24^. The Wombat and Holberg sections and the Calcari con Selce Formation in the Pignola-Abriola and Sasso di Castalda sections are cherty limestones.

### Geochemical evidence of redox conditions at the NRB

Geochemical proxies are used to reconstruct oceanic redox conditions (e.g., V, U, and Mo enrichment)^8,38–43^. Enrichment factors (expressed as X_EF_, see Methods in Supplementary material) for Cr, V, U, and Mo have been widely used to characterize marine redox conditions [e.g^38,44–46^], which generally can be classified as oxic, suboxic, anoxic, or euxinic (presence of free H_2_S)^47^. In this study, these redox-sensitive elements were used to investigate redox conditions across different regions and depths.

Higher concentrations of elements that indicate suboxic to anoxic environments (Cr, V, U, and Mo) were observed in the NRB of the Tethys and eastern Panthalassa oceans (Fig. 1). A slight enrichment of Cr in the Wombat section suggests that at least suboxic conditions existed in the southeastern Tethys Ocean during the NRB interval. Across the NRB, a significant increase in V_EF_ is recorded in sediments deposited in intermediate to deep water environments, represented by the Holberg, Pignola-Abriola and Sasso di Castalda sections (Fig. 1). Vanadium reduction occurs under lower oxygen concentrations^45^. In oxic seawater, V is present as soluble V(V) in the quasi-conservative form of vanadate oxyanions (HVO_4_^2–^ and H_2_VO_4_^2–^). When conditions change from suboxic to weakly anoxic, V(V) converts to V(IV) and forms the vanadyl ion (VO^2+^), related hydroxyl species (VO(OH)^3–^), and insoluble hydroxides (VO(OH)_2_)^48,49^. The highest concentration of V is recorded in the NRB of the Pignola-Abriola section, where V_EF_ values are relatively constant and close to the value of 1 in the Sevatian and abruptly increase across the NRB (Fig. 1). Similar V enrichments are found in the NRB of the Holberg and Sasso di Castalda sections. In the Wombat section, V increases across the NRB, indicating that the shallow marine environment in the southeastern Tethys has changed to a suboxic–anoxic environment (Fig. 1).

On the other hand, the lack of V and U enrichments in the shallow marine sections of Kiritehere apparently does not indicate that these shallow depositional environments might have been oxygen-poor too. However, the Kiritehere section recorded the global extinction of pelagic and benthonic bivalves that belonged to the monitid group, so other mechanisms related to the spread of oxygen-deficient waters might be thus indicated (e.g., ocean acidification, competition, food shortage, eutrophication). In addition, no stratigraphic changes in concentrations of redox-sensitive elements were observed in the deep-sea pelagic deposits of Panthalassa, represented by the Katsuyama section. This suggests that the development of oxygen-poor conditions may not have extended to the deep seafloor of the mid-Panthalassic Ocean (Fig. 1).

**Figure 2:**
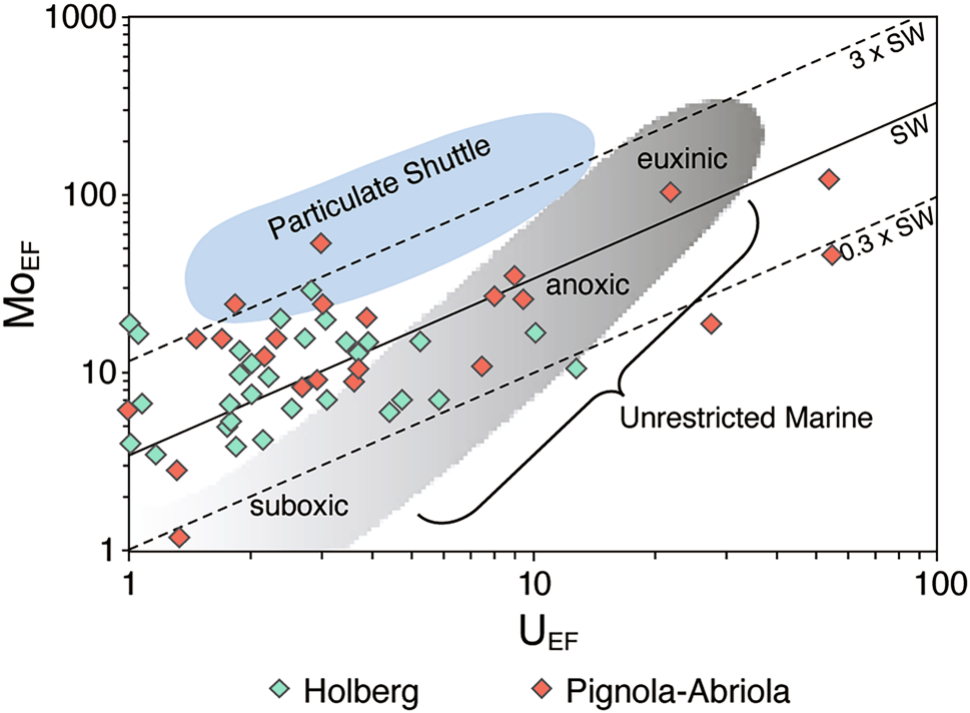
U_EF_ vs. Mo_EF_ diagram for samples in the study sections. Base figure modified from Algeo and Tribovillard^38^.

Another common redox proxy for reducing conditions is the Mo_EF_/U_EF_ ratio, which shows enrichment trends similar to those observed for V_EF_ in both the Pignola-Abriola and Holberg sections (Fig. 1). Covariations of Mo and U enrichment patterns (Fig. 2) and increased Mo_EF_/U_EF_ ratios suggest the development of anoxic bottom water and scavenging of Mo by a Fe–Mn (oxyhydr)oxide particulate shuttle during the upper Sevatian to Rhaetian^38,49^. The occurrence of the particulate shuttle requires an oxic–anoxic redox boundary in the water column and rapid water replacement to maintain the predicted Fe–Mn redox behavior ^38,50^. Thus, the Mo_EF_/U_EF_ increase in the particulate shuttle field in the Holberg and Pignola-Abriola sections may indicate the development of intermediate-depth O_2_ minima with redox boundaries on both sides of Pangea. However, Mo enrichment due to Mn- and Fe- particulate shuttles was less significant compared to modern sites with anoxic oxygen minimum zones (OMZs)^50^. Consequently, V enrichment and U_EF_ and Mo_EF_ trends suggest that anoxic conditions occurred in mid- to deep-water environments over a large area around Pangaea (Fig. 1).

Mo is a conservative hexavalent oxyanion (MoO_4_^2--^) in oxygenated water. The largest sink of Mo in the modern ocean (50%) is adsorption to manganese oxides, which preferentially retains light isotopes. This also occurs in MnO_2_ formed on land. Reporting Mo isotope compositions and correcting published data sets to the NIST 3134 reference solution (δ^98^Mo = ((^98^Mo/^95^Mo)_sample_/(^98^Mo/^95^Mo)_3134_–1) × 1000)^51^, river water δ^98^Mo averages + 0.54‰^52^ and seawater + 2.09‰ ± 0.10^51^, higher than the average continental crust based on molybdenite (0.04‰ ± 1.04) ^53^ or silicate rocks (+ 0.15‰)^54^. As the size of the marine Mn oxide sink of Mo changes over time, depending on the extent of anoxic basins, the δ^98^Mo composition of seawater is expected to also change over time. For example, prior to OAE-2 at 94 Ma, seawater δ^98^Mo was lower (+ 1.65‰)^55^. In conditions where free dissolved sulfide exists at concentrations exceeding 10 mM, Mo is quantitatively removed, and underlying sediments can preserve the isotopic composition of Mo in seawater. Today, euxinic basins are restricted to isolated systems like the Black Sea and fjords where exchange with the open ocean is poor, but in the past they may have been more common. In anoxic but non-euxinic basins, the isotope composition of sedimentary Mo depends on the presence or absence of Mn-oxides and bottom water oxygen. If porewater sulfide is sufficiently close to the sediment–water interface, while bottom water may not be euxinic, seawater Mo can diffuse into the sediment to give them a similar δ^98^Mo to seawater, but the Mo concentration will be lower than in truly euxinic basins^57^. With slightly higher O_2_ in bottom water, but with anoxic pore water, Mn-oxides with low δ^98^Mo that formed within seawater can dissolve in the anoxic sediments, releasing their Mo with low δ^98^Mo. This Mo can diffuse up towards the seawater-sediment interface, or down where it can be trapped permanently in deeper, sulfidic sediments. Today, sediment δ^98^Mo can range from ~ -1‰ in fully oxic systems to + 2.0‰ in euxinic systems, with intermediate values corresponding to redox state and the presence of sulfide. While modern ooids have a composition close to the seawater they precipitate from, use of skeletal carbonates (δ^98^Mo_carb_) as a proxy of seawater can be affected by non-equilibrium (biological) fractionation. Furthermore, δ^98^Mo of impure carbonates will also reflect contributions from detritus and Fe–Mn oxides, which is much lower than the seawater value.

**Figure 3:**
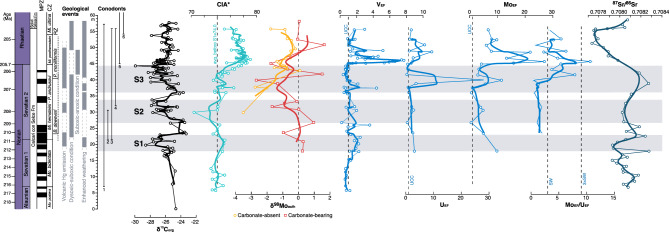
Geochemical profile of Pignola-Abriola section. Enrichment factors (EF) were calculated relative to UCC^58,60^. δ^98^Mo_auth_ for carbonate-absent is of Fe–Mn hydroxides and for carbonate-bearing is Fe–Mn hydroxides plus carbonate. Strontium curve modified after^64^. A description of the taxonomic range of conodonts is as follows: 1. *Mockina bidentata*; 2. *Parvigondolella andrusovi*; 3. *Misikella hernsteini*; 4. *Mi. hernsteini/posthernsteini*; 5. *Mi. posthernsteini;* 6. *Mi. ultima*.

Samples analysed for Mo isotopes were either carbonate-absent (CaO < 1%) or carbonate-bearing. The Mo in carbonate-absent sediments is derived from detritus or authigenic phases, namely Fe–Mn hydroxide and sulfides, while he carbonate-bearing samples also contain significant amounts of potentially isotopically distinct carbonate. Analyses were made on bulk samples, but to address the isotope composition of seawater derived phases, correction for detrital material is made. This is done by assuming all Ti is detrital, and using the upper continental crust (UCC) Mo/Ti composition to determine the amount of detritus-bound Mo. The isotope composition of the UCC is assumed to represent the detrital material, and for this we use UCC Mo concentrations and isotope composition of 1.1 ppm and + 0.1‰^58,59^ to subtract the detrital contribution to the whole rock data by isotope mass balance, leaving a δ^98^Mo value for authigenic (Fe–Mn hydroxide or sulfide) plus carbonate phases. The highest δ^98^Mo in carbonate will be close to seawater, and our highest δ^98^Mo_carb_ of + 1.65 is similar to that of Cretaceous seawater suggesting that Late Triassic seawater was somewhat similar^55^. In the Pignola-Abriola section, the δ^98^Mo in carbonate-absent samples increases from -3.5‰ at 30 m to just 0.1‰ at 42.9 m, while the δ^98^Mo in carbonate-bearing samples are more variable (-2.7‰ to + 1.7‰) (Fig. 3).

In an oxic water column, Fe–Mn hydroxides are 3‰ lower in δ^98^Mo than seawater^56^, but the lowest values of δ^98^Mo we measure are more than 3‰ lower than our estimate of seawater at the time of formation. To increase this difference beyond 3‰, dissolution of Fe–Mn hydroxides (bearing low δ^98^Mo signatures) followed by their re-oxidation is indicated, suggesting that the oxygen content of water close to the seawater sediment interface fluctuated in response to physical restriction, which is unlikely given the paleo-reconstruction of the Pignola-Abriola area, or to changes in the delivery of organic matter, supported by the correspondence between the lowest δ^98^Mo and highest TOC%. The increase in δ^98^Mo_auth_ from values consistent with a mostly, albeit variable, oxic water column to values consistent with anoxic to euxinic sediments underlying an expanding area of high productivity with a persistent oxygen minimum zone^61^. The increase in δ^98^Mo_auth_ towards that of contemporaneous seawater indicated by the highest δ^98^Mo_carb_ as Mo_EF_/U_EF_ also increases supports the concept of intensification of an OMZ driven by increasing productivity, and the progression of a porewater sulfide front towards the sediment–seawater interface.

### Possible cause(s)

**Figure 4:**
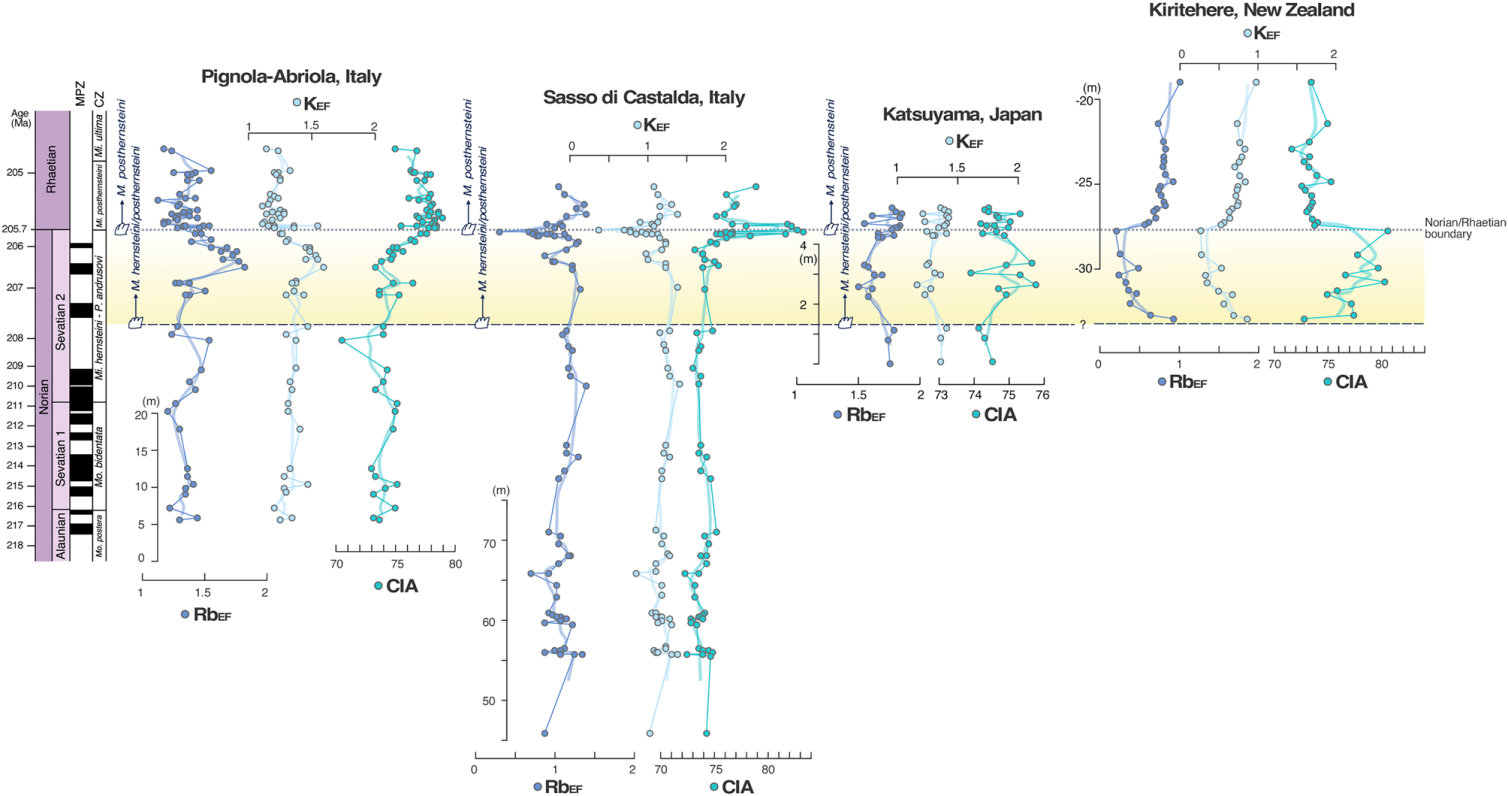
Stratigraphic profiles of K_EF_, Rb_EF_, and CIA in the Pignola-Abriola section. Enrichment factors (EF) were calculated relative to UCC^60^. K_EF_ and Rb_EF_ decrease with increasing CIA values across the NRB.

These environmental perturbations can be explained by various mechanisms, any of which may create a global record, such as dissociation of clathrate hydrate and/or enhanced magmatic activity and outgassing^1,62,63^. Likely, a large volume of ^13^C-depleted CO_2_ entered the ocean–atmosphere system prior to the NRB, possibly as a product of volcanic outgassing by the emplacement of the Angayucham LIP^11,21,24,32,64^. This hypothesis is supported by the comparison of C-isotope records from marine^11^ and terrestrial settings^21,22^ with the seawater Sr-isotope^65^ and Os-isotope curves^66–68^. The ^87^Sr/^86^Sr and ^187^Os ⁄^188^Os values started to decline in the upper Norian, indicating a relative increase in the supply of mantle-derived unradiogenic Sr and Os from marine hydrothermal or other mafic igneous sources. Furthermore, the mechanism invoked to explain the spread of reducing conditions also implies significant input of CO_2_ into the ocean–atmosphere system that would enhance chemical weathering via acceleration of the hydrological cycle during radiative-forced warming and cause increased nutrient discharge (e.g., nitrates and phosphates) to the global ocean^1,69,70^. The coincidence of these three concatenated steps has been clearly documented worldwide. Our weathering proxy data (Chemical Index of Alteration—CIA, Rb_EF_, and K_EF_ values) have revealed that chemical weathering intensified during the development of anoxic conditions (Figs. 3, 4).

Values of the CIA from the shale samples^71^ indicate the extent of decomposition of feldspar minerals, which are the most abundant mineral group in the UCC. Because of the high carbonate content of the study section, we used a modified form of the CIA equation by^33^ as:

$${\text{CIA}}^* = {\text{Al}}_{2} {\text{O}}_{3} /\left( {{\text{Al}}_{2} {\text{O}}_{3} + {\text{Na}}_{2} {\text{O}} + {\text{K}}_{2} {\text{O}}} \right)/100,$$

CIA values are relatively constant below the FO level of *M. posthernsteini* s.l. sensu^34^, and then rapidly increase in the S3 interval (sensu^32^, Fig. 3). These stratigraphic variations in CIA values indicate intensified chemical weathering on continents during the S3 interval, implying that the climate changed to relatively warm and humid conditions. K and Rb were released from primary minerals, possibly micas and feldspars in felsic rocks, during continental chemical weathering. The stratigraphic variations in K_EF_ and Rb_EF_ decrease across the NRB, associated with a rapid increase in CIA values (Figs. 3, 4), which suggest the intensity of aluminosilicate chemical weathering increased during the anoxic event.

In theory, enhanced chemical weathering via acceleration of the hydrological cycle would cause increased nutrient discharge to the global ocean, with a consequent increase in biological productivity^1,70,72^. Barium is commonly used as a tracer of paleo-productivity^73–76^, because barite precipitation occurs in decaying particulate organic matter while it sinks to the seafloor^74,77,78^, which is supported by enhanced Ba_EF_ values below high-productivity areas^79^. Our data show that Ba_EF_ increased in the NRB, except in the Panthalassic Katsuyama section, deposited below the CCD (Fig. 1). Hence, our geochemical proxies show an apparent link between the volcanic event and oceanic anoxia via increased chemical weathering and paleo-productivity during the S3 interval.

High CIA continues after the boundary, indicating the persistence on land of the conditions that triggered the OAE even as the ocean began to recover, first exhibited by Mo_EF_/U_EF_^50^. δ^98^Mo_auth_ also begins to record increasing oxic conditions well after the boundary, showing the control that Mn has on δ^98^Mo. The prolonged chemical weathering could be the result of a million-year timescale required for silicate weathering to consume the massive amounts of CO_2_ emitted during volcanism. Long-term increases in CIA similar to the NRB have also been reported from the early Late Triassic Carnian Pluvial Episode (CPE; 232–234 Ma) and from the Triassic-Jurassic boundary^80,81^. In the case of the Triassic-Jurassic boundary, it is estimated that 1–2 myr were necessary for chemical weathering^80^ to consume the large amount of CO_2_ released by 600 kyr of volcanic activity^82^. CO_2_ emissions from voluminous, pulsed volcanic activity across the NRB may have thus caused prolonged continental weathering in the Rhaetian.

In summary, our data document a previously unknown OAE of global extent, similar in nature to other OAEs. The OAE that spanned the NRB extended across the Panthalassa Ocean to both sides of the Pangea supercontinent and it is recorded in both the Northern and Southern Hemispheres, at different latitudes. As is the case for most OAEs, we propose that this Norian-Rhaetian oceanic oxygen depletion event resulted from large-volume emissions of volcanogenic greenhouse gases that accelerated the hydrological cycle, thereby increasing weathering rates and increasing nutrient delivery to the oceans. The increased nutrient supply enhanced primary productivity and export of organic carbon, resulting in oxygen depletion and expansion of the OMZ, which in turn increased the burial efficiency of organic carbon in sediments. Fossil extinctions and reducing conditions were documented in basins around the world (Italy, Canada, NW Australia, New Zealand), leaving an unmistakable record of the NRB OAE. The onset of the stepwise Late Triassic extinctions coincided with the NRB OAE, indicating that the combined climate and environmental changes impacted biota at this time. The trigger of this event is attributed to a volcanic event pre-dating the NRB or an alternative source of volcanogenic gas emissions, likely from the Angayucham LIP.

### Supplementary Information

Supplementary Information.

Supplementary Tables.

## Data Availability

All data generated or analyzed during this study are included in this published article and supplementary information files.
