# Supplementary material for: Unveiling a new oceanic anoxic event at the Norian/Rhaetian boundary (Late Triassic)
Source: Sci Rep. 2024 Jul 6;14:15574. doi: 10.1038/s41598-024-66343-z (PMC11227520; doi:10.1038/s41598-024-66343-z)
Supplement: Supplementary file 1 — Supplementary Information. [file 41598_2024_66343_MOESM1_ESM.docx]

**SUPPLEMENTARY MATERIAL**

Unveiling a new Oceanic Anoxic Event at the Norian/Rhaetian Boundary (Late Triassic)

**Authors:** Manuel Rigo^1,2,*^, Xin Jin^3^, Linda Godfrey^4^, Miriam E. Katz^4,5^, Honami Sato^6^, Yuki Tomimatsu^6^, Mariachiara Zaffani^1^, Matteo Maron^7^, Sara Satolli^7^, Giuseppe Concheri^8,^ Alessandra Cardinali^8^, Qiangwang Wu^1^, Yixing Du^3^, Jerry Zhen Xiao Lei^9^, Connor S. van Wieren^9^, Lydia S. Tackett^10^, Hamish Campbell^11^, Angela Bertinelli^12^, Tetsuji Onoue^6^

**Affiliations:**

^1^ Department of Geosciences, University of Padova, Via G. Gradenigo 6, 35131 Padova, Italy

^2^ IGG-CNR (Istituto di Geoscienze e Georisorse), Padova, Firenze

^3^ State Key Laboratory of Oil and Gas Reservoir Geology and Exploitation and Key Laboratory of Deep-time Geography and Environment Reconstruction and Applications of Ministry of Natural Resources, Chengdu University of Technology, Chengdu 610059, China

^4^ Department Earth and Planetary Sciences, Rutgers University, Piscataway, NJ 08854, USA

^5^ Geosciences Dept., Union College, Schenectady, NY 12308

^6^ Department of Earth and Planetary Sciences, Kyushu University, Fukuoka, 819-0395 Japan.

^7^ Department of Engineering and Geology, University “G. d’Annunzio” of Chieti-Pescara, Via dei Vestini 31, 66100 Chieti, Italy

^8^ Department of Agronomy Food Natural Resources Animals and Environment (DAFNAE), University of Padova, Viale dell'Università, 16, 35020 Legnaro, Italy

^9^ School of Earth and Ocean Sciences, University of Victoria, 3800 Finnerty Road, Bob Wright Centre A405. Victoria, British Columbia, Canada V8P 5C2

^10^ Department of Geological Sciences, University of Missouri, Geological Sciences Bldg, 101, 400 S 6th St, Columbia, MO 65201, USA

^11^ GNS Science, 1 Fairway Drive, 5010 Lower Hutt, Wellington, New Zealand

^12^Departmento of Physics and Geology, University of Perugia, Via A. Pascoli, 06123, Italy

*Correspondence to: [manuel.rigo@unipd.it](mailto:manuel.rigo@unipd.it)

**Geological setting of the studied sections**

*1. Lagonegro Basin*

The Lagonegro Basin is located in the Southern Apennines (Italy) and is considered as an oceanic basin of the western Tethys (Stampfli et al., 2003; Ciarapica and Passeri, 2005). In fact, the Lagonegro sedimentary succession lies upon an oceanic crust as illustrated by the available seismic lines and on the magnetic anomaly pattern of floor of the Ionian Sea (Finetti, 2003; Catalano et al., 2001; Argnani, 2005; Rigo et al, 2007, 2012). The Lagonegro sedimentary succession ranges from Permian to Miocene and it consists of shallow to deep basinal pelagic successions (Scandone, 1967; Rigo et al., 2005, 2012; Giordano et al., 2010). The Upper Triassic is represented by the Calcari con Selce (Formation), consisting of limestones with chert nodules rich in conodonts, radiolarians and thin-shelled pelagic bivalves (e.g. *Halobia, Monotis*) intercalated with marls, shales and calcarenites, and the overlying Scisti Silicei (Formation) that are cherts and radiolarites (Rigo et al., 2005, 2012; Reggiani et al., 2005, Bazzuchi et al., 2005, Bertinelli et al., 2005, 2016; Giordano et al., 2010, 2011). The so-called “transitional interval” (Amodeo, 1999; Rigo et al., 2012) between these 2 formations represents the beginning of biosiliceous sedimentation and it is considered diachronous, ranging from uppermost Norian to lower Hettangian (Bertinelli et al., 2005; Reggiani et al., 2005; Giordano et al., 2010, 2011). The base of the “transitional interval” consists of a 3 meter-thick interval of red shale (Amodeo, 1999; Bertinelli et al., 2005; Rigo et al., 2005, 2012; Giordano et al., 2010) and it is Sevatian 1 in age (base of the *Mockina bidentata* Zone) (Reggiani et al., 2005).

*1.1. Pignola-Abriola section*

The Pignola-Abriola section crops out on the mountainside of Mt. Crocetta (Geographic coordinate system, datum WGS 84: 40° 330 23.50″N, 15° 470 1.71″E), close to the village of Pignola (Potenza, southern Italy). This section consists of the upper part of the Calcari con Selce, in which the Norian-Rhaetian transition is well documented (Amodeo, 1999; Bazzucchi et al., 2005; Rigo et al., 2005, 2016; Giordano et al., 2010). The basal part (from 0 to 13 m) of the Pignola-Abriola section consists of thin-bedded, cherty limestones (sometimes dolomitized) intercalated with thin layers of shale and calcarenite. The overlying 37 meters are characterized by a progressive decrease in the relative abundance of carbonates in favor of siliceous components (Amodeo, 1999; Bazzucchi et al., 2005; Giordano et al., 2010) and consists of limestones (sometimes silicified) alternating with dark grey shales and thin beds of black chert. The NRB interval is instead shaley and represented by repetitive, thin and well-laminated interbeds of black shale, suggesting a transient period from dysoxic to more oxic towards anoxic conditions (Casacci et al., 2016). This section belongs to the intermediate facies association (Scandone, 1967; Giordano et al., 2010; Casacci et al., 2016).

The Pignola-Abriola section is well biostratigraphically well constrained, yielding rich assemblages of conodonts and (mostly pyritized) radiolarians (Bazzucchi et al., 2005; Rigo et al., 2005, 2016). At ca. meter 7 above the base, *Mockina bidentata* first occurs (Giordano et al., 2010), defining the base of the *M. bidentata* Zone (Kozur and Mock, 1991; Rigo et al., 2018) and the base of the Sevatian. At meter 21.4, the lowest occurence (LO) of *Misikella hernsteini* defines the base of the *M. hernsteini* Zone, Sevatian 2 (Rigo et al., 2016, 2018). At ca. 32 meters, the first representative of a *M. hernsteini/posthernsteini* transitional form is observed, and at 44.9 meters *Misikella posthernsteini* appears, the first occurrence of which marks the base of the Rhaetian stage and defines the base of the eponymous conodont biozone (Kozur and Mock, 1991; Rigo et al., 2016, 2018). *Misikella ultima* occurs at ca. 54.2 meters (Rigo et al., 2016). Conodonts have a CAI (conodont alteration index) ≤1.5 (Giordano et al., 2010; Rigo et al., 2016). The radiolarians collected are generally pyritized, though not always well preserved, but still useful for radiolarian biostratigraphy: at ca. meter 22, the base of the *Betraccium deweveri* Assemblage Zone (Carter, 1993*)* was documented, followed by the base of the *Proparvicingula moniliformis* Assemblage Zone at 41 meters.

*1.2. Sasso di Castalda section*

The Sasso di Castalda section crops out near the homonymous village, along the SW side of the Monte Buccaglione (Geographic coordinate system, datum WGS 84: 40° 29’ 29.47″N, 15° 40’ 33.78″E). This section consists of 110 m of gray mud limestones with list and nodules of cherts intercalated by thin shales (Calcari con Selce). The limestones yield rich associations of conodonts, radiolarians and thin bivalves (i.e. *Halobia*). The age of the section is from middle Norian (Alaunian) to Rhaetian, and the base of “transitional interval” is well marked by the 3 m thick red level which is Sevatian in age (Bertinelli et al., 2005; Rigo et al., 2005, 2012), while the Norian/Rhaetian boundary is documented at the base of the Scisti Silicei with integrated conodont and radiolarian biostratigraphy, the main events of which are the FO of *Misikella posthernsteini* and the base of the radiolarian *Proparvicingula moniliformis* Zone. Just below the Norian/Rhaetian boundary, the evidence of the Rochechouart impact event has beed recently documented (Sato et al., 2021)

*2. Wombat Basin, northwestern Australia, ODP SITE 761C*

Site 761 is located in the northern Wombat Basin (Geographic coordinate system, datum WGS 84:16°44.23'S, 115°32.10'E), at a water depth of ca. 2167.9 meters. It records an almost continue Upper Triassic to Quaternary succession. The Triassic portion of Hole 761C is dominated by calcareous layers, subdivided by their microfossils, reflecting a series of environmental changes. The lowermost interval (core 33R) consists of clayey siltstone, late Norian age defined by the dinoflagellate cysts *Heibergella balmei* and nannofossils (Bralower et al., 1992). The overlying interval (cores 32R up to 30R) shows upwards an increase of the carbonate content, and the occurrence of the nannofossil *Thoracosphaera geometrica* and foram *Triasina oberhauseri* both dating this interval as Norian (Bralower et al., 1992; Zaninetti et al., 1992). Gardin et al. (2012) re-analysed the material from Hole 761C, documented the typical Rhaetian nannofossil *Euconusphaera zlambachensis* in core 32R3, placing the NRB in core 32R (417.7–421.9 mbsf). Ostracod and dinoflagellate cyst biostratigraphic studies also suggest that the NRB is located in the core 32R (Brenner et al., 1992). Between 33R1 and 32R2, the significant negative δ^13^C_org_ at the Norian/Rhaetian boundary was documented (Rigo et al., 2020).

*3. Murihiku Terrane - Zealandia (New Zealand), Kiritehere section*

The ‘Kiritehere section’ is located on the Kiritehere beach, on the western Tasman Sea coast of the central North Island of New Zealand, close to village of Marokopa (Geographic coordinate system, datum WGS 84: 38°31.16'S, 174°71.01'E ) (Grant-Mackie, 1981; Rigo and Campbell, 2022). The rocky shore platform at Kiritehere is well-exposed and consists of volcaniclastic sedimentary rocks dominated by fine sandstone and siltstone, with minor but conspicuous conglomerates, tuffs and shell beds (Arawi Shellbeds and Ngutunui formations)(Grant-Mackie, 2015, Rigo et al., 2020; Rigo and Campbell, 2022). The formations are fossiliferous, especially the *Monotis*-bearing Arawi Shellbeds, while the Ngutunui Formation bears few small size bivalves (Grant-Mackie, 1981, 2013). Other fossils within both the Arawi Shellbeds and the Ngutunui Formation include bivalve of genera *Kalentera*, *Lima* and *Maoritrigonia*, and of brachiopod belonging to genera *Clavigera*, *Rastelligera, Psioidiella, Mentzelia* and *Sakawairynchia*, and other rare gastropods, cephalopods and crinoids (Grant-Mackie, 1981, 2013). Recently, the global δ^13^C_org_ negative shift documented at the Norian/Rhaetian boundary was recorded in the lower part of the Ngutunui Formation (Rigo et al., 2020; Rigo and Campbell, 2022).

*5. British Columbia - Canada, Holberg section: 50.632056°N, 127.959509°W*

Vancouver Island is situated within the Insular Belt of the Canadian Cordillera (e.g. Muller, 1977), and represents the most outboard layer in a series of allochthonous tectonostratigraphic terranes that sequentially accreted onto the western margin of North America (e.g. Monger & Ross, 1971; Monger, 1997). The geology of Vancouver Island is specifically of Wrangell Terrane affinity (e.g. Jones et al., 1977), an expansive terrane characterized by a consistent Permian through Triassic sequence capped by Upper Triassic carbonate overlying thick basaltic flows forming an oceanic plateau (e.g. Jones et al., 1977). Several corroborating lines of evidence suggest this Upper Triassic carbonate experienced significant northward movement following deposition and prior to accretion with North America (e.g. Hillhouse & Gromme, 1984; Aberhan, 1999). The Carnian Quatsino Fm and Norian Parson Bay Fm represent the carbonates of this sequence on Vancouver Island (e.g. Nixon & Orr, 2007). The Parson Bay Fm is predominantly comprised of an interbedded carbonate-siliciclastic unit, with a comparatively minor volcanic unit (e.g. Nixon & Orr, 2007). The siliciclastic beds are mostly shale and siltstone, but can include sandstone and even conglomerate (e.g. Nixon & Orr, 2007). The wide variety lithologies present and sedimentary structures observed suggests the Parson Bay Fm represents a depositional environment ranging from shallow/moderate depth marine, to high-energy intertidal (e.g. Nixon & Orr, 2007). The Parson Bay Fm specifically as exposed along the southern shore of the westernmost portion of Holberg Inlet can be divided into 2 lithologically distinct portions. The lower portion is comprised primarily of 5–25cm interbedded and dark laminated calcareous shale and wackestone with pervasive *Monotis* sp. death assemblage layers (bivalve diameter ~2–10cm, no clear size gradient with time). These bivalves tend to be preserved intact and detailed, but highly compressed. Thicker grainstone and mudstone beds are also present, as well as a single volcanic basalt bed. The upper portion is more siliciclastic and less calcareous, comprised of bedded, highly silt-rich grainstone. The last occurrence (LO) of *Monotis* sp. is observed 12.6 m below the transition from the lower portion to the upper portion, at 277.8 m above the base of the section. Therefore, the NRB interval for this study was tentatively placed between the LO of *Monotis* (277.8 m) and a negative δ^13^C_org_ excursion at 284.75 m above the base of the section.

This section was investigated for δ^13^C_org_, and 49 samples were first cleaned, dried and then smashed in powder before being attacked with HCl 10% overnight, and then analyzed in the Department of Geosciences, University of Padova (Italy) by using a Delta V Advantage mass spectrometer connected to a Flash HT Elemental Analyzer. For every set of analyses, multiple blank capsules and isotope standards (IAEA CH-7 = -32.15‰, IAEA CH-6 = -10.45‰) (Coplen et al., 2006) were included. The standard deviation of the in-house standard (δ^13^C_org_ = -26.00‰) was better than 0.2‰ during the period of analyses. The resulting δ^13^C_org_ profile first shows a negative trend from the base of the section up to meter 290, followed by an increase of the δ^13^C_org_ curve towards the background values.

*6. Japan, Katsuyama section*

Samples for biostratigraphic and geochemical investigations were collected from chert and shale layers from the Katsuyama section (35°25′21.6′′N, 136°58′16.5′′E), Mino Belt, central Japan (Carter and Hori, 2005; Fujisaki et al., 2018). The Mino Belt is an accretionary complex generated during the Middle–Late Jurassic subduction (Matsuda and Isozaki, 1991). The succession was deposited below the CCD and consists of bedded cherts, shales and radiolarites, representing a pelagic and open ocean setting located in the low latitudes of the Panthalassa Ocean (Ando et al., 2001; Uno et al., 2015). The studied Katsuyama section is 6 m thick and consists in bedded red chert and shales. Based on radiolarian and conodont biostratigraphic investigation from bedded cherts, the NRB was places with the FO of the conodonts *Misikella posthernsteini* (Supplementary Fig. S5).

Shales were investigated for C isotopes from organic matter. Twenty-three samples were thus cleaned with deionized water, dried, and then pulverized before being attacked with HCl at 10% overnight. After the neutralization of the acid solution, the samples were dried and analyzed three times along blank capsule, in-house and international standards ((IAEA CH-6 = −10.45‰, IAEA CH- 7 = −32.15‰, Coplen et al., 2006) with a Delta V Advantage mass spectrometer connected to a Flash HT Elemental Analyzer via CONFLO at the Department of Geosciences, University of Padova (Italy).The sample values of each sample are reported in the Supplementary table S7 and are illustrated in Fig S5. The resulting profile of δ^13^C_org_ analyses shows in stratigraphic order a first positive peak of ca. 1.5‰ from the background values, followed by negative trend that reached the most negative peak (ca 2‰) just below the first occurrence of *M. posthernsteini*, before returning to background values. The δ^13^C_org_ profile of Katsuyama section is very consistent with the coeval C organic curves documented in other regions (Rigo et al., 2020).

**Material and Methods**

***TOC***

The organic carbon content in rock samples was measured by an ignition method using Skalar Primacs ATC 100-IC-E (Skalar PrimacsSNC-100, Skalar Analytical BV, Breda, The Netherlands). Skalar Primacs uses a measuring principle in accordance with DIN 19539: 100 mg of rock powder are gradually brought from 150 to 400 °C in 480 s, with a controlled temperature increase of 70 ° C per minute. An IR detection system of the emitted CO_2_ flow returns the measurement of the carbon lost in percentage content with respect to the weight of the sample. The reproducibility of this method was first tested measuring 12 standards 3 times each, and the resulting deviation standard ranges between 0,02% and 0,25% (Fig. S1). The reliability of this method was also tested comparing the TOC results of 7 random samples from Pignola-Abriola and Kiritehere sections measured with both Skalar and EA- IRMS (Elemental Analyzer - Isotope Ratio Mass Spectrometry). The samples consisted of shales, marls and limestones that were crushed and reduced into powder and after being weighed in silver capsule, they were attacked overnight by a 10% HCl acid and then measured 3 times by using Delta V Advantage mass spectrometer, the deviation standard of which ranges between 0.00% and 0.04%. These samples have been analysed twice with Skalar, showing a reproducibility ranging between 0.00% and 0.02%, which is similar or better than those achieved by measuring the 12 standards (0,02%-0,25%). The results from the rock samples between the 2 methods are fully comparable and the results are listed in Fig. S1.

Fig. S1. TOC analyses and reproducibility of STD samples by using Skalar (A), and comparison between the Skalar and Delta V EA-IRMS TOC results (B).

***Major, trace and rare earth element analysis***

After drying the powder samples at 60˚C, the samples were pressed at 2×10^4^ kg for 3 min to yield pressed powder pellets. Major and some trace element (Cr, Ni, Cu, Zn) concentrations were determined by X-ray fluorescence spectrometry (XRF: PANalytical Epsilon 3XLE with a Mo X–ray tube) on pressed powder pellets at Kyushu University, Japan. Samples were calibrated using 21 standard rock samples issued by the Geological Survey of Japan. Reproducibility, based on repeat analyses of four standards (JSd-1), was better than ±0.5% for Mg, Al, Si, K, Ca, Ti, and Fe; better than ±1% for Na and Mn; and better than ±10% for Cr, Ni, Cu and Zn. Detection limits for trace elements were 4 ppm for Ni, 3 ppm for Cr and Cu, and 2 ppm for Zn. In addition, the bulk chemical composition of 28 samples from the Pignola-Abriola section was determined by XRF using a WDS sequential Philips PW2400 spectrometer equipped with a 3 kW Rh X-ray tube, 4 filters (Al 200 μm, Brass 100 μm, Pb 1000 μm and Brass 300 μm), 3 collimators (150 μm, 300 μm and 700 μm), 5 analyzing crystals (LiF220, LiF200, Ge111, PE002, TlAP100), 2 detectors (flow counter and scintillator), and the sample changer Philips PW2510 with 30 sample holders. The analyses, performed under vacuum conditions, and using the SuperQ software from Panalytical, were based on calibrations calculated on geological reference standards (Govindaraju, 1994). The samples were crushed in an agate mortar, and the resulting powders were first used to determine the loss on ignition (LOI) heating them in a furnace at 860°C for 20 minutes, and then at 980°C for 2 hours. The calcined powders were then diluted with flux di-lithium tetraborate Li_2_B_4_O_7_ (1:10 ratio) and melted with a fluxer Claisse Fluxy (reaching a temperature of about 1150°C) to obtain glass beads for XRF analyses. In order to include the LOI value (expressed as %) into the sum of major element oxides, analyses were normalised to 100% minus LOI value. Instrumental precision (defined by several measurements performed on the same sample) is within 0.6% relative for major and minor elements, and within 3% relative for trace elements. The XRF accuracy was checked by reference standards (Govindaraju, 1994) and was within 0.5 wt% for Si, lower than 3% for other major and minor elements, and lower than 5% for trace elements. The lowest detection limits of XRF were within 0.02 wt% for Al_2_O_3_, MgO and Na_2_O, within 0.4 wt% for SiO2, within 0.005 wt% for TiO_2_, Fe_2_O_3_, MnO, CaO, K_2_O and P_2_O_5_ and within a range between 3 and 10 ppm for trace elements.

Furthermore, 285 samples were analysed for trace and rare earth element (REE) concentrations using inductively coupled plasma–mass spectroscopy (ICP–MS) with lithium metaborate/tetraborate fusion at Actlabs (package Code 4B2-STD), Ancaster, Canada. Ten international rock and mineral standards were also analysed for quality control during trace and REE analyses. For details of the analytical techniques, see www.actlabs.com.

***δ^98^Mo - Molybdenum isotopes***

δ^98^Mo was determined using the double spike method using ^97^Mo and ^100^Mo in proportions according to Rudge et al.^19^ and reported relative to NIST 3134^20^. Samples were weighed into PFA vials and spike added to give ideal sample-spike ratios and then digested using different mineral acids (HF, HNO_3_, and HCl) with hydrogen peroxide added to ensure that Mo was in the Mo(VI) oxidation state. Once the samples had fully dissolved and the spike fully equilibrated, the samples were dried and dissolved in a 1N HF/0.5N HCl acid mixture and loaded on 2mL Bio-Rad AG 1-X8 resin following the single column method described in Pearce et al.^21^, the only difference being the elution of Mo in 7N nitric acid, which avoids co-elution of U which we found may not fully be eluted using 4N HCl.

Sample solutions were dissolved in 2% HNO_3_/0.1% HF introduced at 50 μL/min into a ThermoScientific Neptune Plus MC ICPMS using a Cetac Aridus II at Rutgers University at concentrations around 50 ppb. Ni sample cone and an X-profile Ni skimmer cone were used and the seven Mo isotopes were collected in low resolution mode and static mode using 10^11^-ohm resistors in those amplifiers. ^91^Zr and ^99^Ru were also monitored. Sample uptake time was 90s including the time for on peak 30s baselines. Sample rinse times were 5 minutes. 5 blocks of 20 cycles (100 ratios) were collected with 4s integration times for each cycle with a 3 s idle time. Typical signal sensitivity was around 200V/ppm. Acid blanks which were less than 2 mV were analysed between each sample and subtracted offline prior to the double spike inversion. Molybdenum NIST standard 3134 and an in-house Johnson Matthey standard (JM) were analysed periodically during each collection period as were seawater and USGS standard SDO. NIST 3134 returned a value of -0.05±0.04‰ (2SD, n=9) and was used to correct other standards and unknowns. Over a 3 year period standards JM returned a δ^98^Mo value of -0.19±0.06‰ (2SD, n=17), surface N Atlantic seawater +2.01±0.06‰ (2SD, n=5) and SDO +0.79±0.04‰ (2SD, n=7).

***Calculation of enrichment factors***

Samples from the Wombat and Holberg sections and the Calcari con Selce Fm. (i.d. cherty limestones) in the Pignola-Abriola and Sasso di Castalda sections are rich in calcium carbonate, and the abundance of major and trace elements are negatively correlated with CaO content Sato et al., 2021; Maron et al., 2024). This feature is attributed to dilution by biogenic carbonate, as illustrated by the CaO–Al_2_O_3_, CaO –K_2_O, and CaO –TiO_2_ plots (Fig. S1). A similar relationship can be found in the bivariate plots of SiO_2_ versus terrigenous elements in the Katsuyama section and the the Scisti Silicei Fm. (i.e. cherts and radiolarites) in the Pignola-Abriola and Sasso di Castalda sections (Fig. S1). Consequently, redox sensitive elements and other trace elements in the studied samples are largely diluted by biogenic carbonates or silica (e.g., radiolarians).

**Fig. S1 - Effect of dilution by biogenic Si and Ca. Plots of SiO_2_, CaO vs Al_2_O_3_, K_2_O, and TiO_2_ for samples in the study sections.**

Dilution by biogenic components can increase relative errors of compositional data and obscure their in-situ enrichment. To compensate for this, elemental concentrations were normalized using Ti concentrations and compared with those of upper continental crust (UCC; McLennan, 2001) to obtain enrichment factors. The enrichment factor (X_EF_) is defined as follows:

X_EF_ = (X_sample_ / Ti_sample_) / (X_UCC_ / Ti_UCC_), (1)

where X and Ti are the weight concentrations of element X and Ti, respectively. Samples with TiO_2_ concentrations below the limit of quantitation (TiO_2_ < 0.02-0.06 %) were not included in the EF calculations.

Values of the Chemical Index of Alteration (CIA) from the shale samples (Nesbitt and Young, 1982) indicate the extent of decomposition of feldspar minerals, which are the most abundant mineral group in the UCC. Because of the high carbonate content of the study section, we used a modified form of the CIA equation by Casacci et al. (2016) as:

CIA* = A1_2_O_3_ / (A1_2_O_3_ + Na_2_O + K_2_O) / 100

**References**

Aberhan, M. (1999). Terrane history of the Canadian Cordillera: Estimating amounts of latitudinal displacement and rotation of Wrangellia and Stikinia. Geological Magazine, v. 136, p. 481–492.

Amodeo, F., 1999. Il Triassico terminale–Giurassico del Bacino Lagonegrese: Studi stra- tigrafici sugli Scisti Silicei della Basilicata (Italia meridionale). Lausanne, Mem. Geol. 33, 1–123.

Ando A, Kodama K, Kojima S. 2001. Low-latitude and Southern Hemisphere origin of Anisian (Triassic) bedded chert in the Inuyama area, Mino terrane, central Japan. *Journal of Geophysical Research-Solid Earth* 106, 1973-1986.

Argnani, A., 2005. Possible record of a Triassic ocean in the Southern Apennines. Boll. Soc. Geol. Ital. 124, 109–121.

Bazzucchi, P., Bertinelli, A., Ciarapica, G., Marcucci, M., Passeri, L., Rigo, M., Roghi, G., 2005. The Late Triassic–Jurassic stratigraphic succession of Pignola (Lagonegro- Molise Basin, Southern Apennines, Italy). Boll. Soc. Geol. It. 124, 143–153.

Bertinelli, A., Ciarapica, G., De Zanche, V., Marcucci, M., Mietto, P., Passeri, L., Rigo, M., Roghi, G., 2005. Stratigraphic evolution of the Triassic–Jurassic Sasso di Castalda succession (Lagonegro basin, Southern Apennines, Italy). Boll. Soc. Geol. It. 124, 177–188.

Bralower, T.J., Bown, P.R., Siesser, W.G., 1992. Upper Triassic calcareous nannoplankton biostratigraphy, Wombat Plateau, northwest Australia. Proc. Ocean Drill. Program Sci. Results 122, 437–451.

Carter ES, Hori RS. 2005. Global correlation of the radiolarian faunal change across the Triassic-Jurassic boundary. *Canadian Journal of Earth Sciences* 42, 777-790.

Carter, E.S., 1993. Biochronology and Paleontology of uppermost Triassic (Rhaetian) radiolarians, Queen Charlotte Islands, British Columbia, Canada. Lausanne, Switzerland, Mem. Geol. 11, 1–176.

Casacci, M., Bertinelli, A., Algeo, T.J., Rigo, M., 2016. Carbonate to biosilica transition at the Norian-Rhaetian boundary controlled by rift-related subsidence in the western Tethyan Lagonegro Basin (southern Italy). Palaeogeogr. Palaeoclimatol. Palaeoecol. 456, 21–36.

Catalano, R., Doglioni, C., Merlini, S., 2001. On the Mesozoic Ionian Basin. Geophys. J. Int. 144, 49–64.

Ciarapica, G. & Passeri, L. 2005: Ionian tethydes in the Southern Apennines. In Finetti, I.R. (ed): Crop Project: Deep Seismic Exploration of the Central Mediterranean and Italy, 209–224. Elsevier, Amsterdam.

Coplen, T.B., Brand, W.A., Gehre, M., Groning, M., Meijer, H.A.J., Toman, B., Verkouteren, R.M., 2006. New guidelines for δ13C measures. Anal. Chem. 78, 2439–2441.

Finetti I.R. (2003) - *Depositional basin and tectonodynamics of the «Ionides» (Lagonegrese units) from new CROP seismic data*. Int. Workshop «Late Triassic-Early Jurassic events in the frame- work of the Pangea break-up», Capri, 30 Sept.-1 Oct. 2003, Abstracts, 36-37.

Fujisaki W, Matsui Y, Asanuma H, Sawaki Y, Suzuki K, Maruyama S. 2018. Global perturbations of carbon cycle during the Triassic–Jurassic transition recorded in the mid-Panthalassa. *Earth Planet Sci Lett* 500, 105-116.

Gardin, S., Krystyn, L., Richoz, S., Bartolini, A., Galbrun, B., 2012. Where and when the earliest coccolithophores? Lethaia 45, 507–523.

Giordano, N., Ciarapica, G., Bertinelli, A., Rigo, M., 2011. The Norian–Rhaetian interval in two sections of the Lagonegro area. The transition from carbonate to siliceous deposition. Ital. J. Geosci. 130, 380–393.

Giordano, N., Rigo, M., Ciarapica, G., Bertinelli, A., 2010. New biostratigraphical con- straints for the Norian/Rhaetian boundary: Data from Lagonegro Basin, Southern Apennines, Italy. Lethaia 43, 573–586. https://doi.org/10.1111/j.1502-3931.2010. 00219.x.

Govindaraju, K. 1994. Compilation of working values and sample description for 383 geostandards. Geo- standards Newsletter, 18, 1-158.

Grant-Mackie, J.A., 1981. New Zealand Warepan (Upper Triassic) sequences: Murihiku Supergroup of the North Island. J. R. Soc. New Zeal. 11, 31–56.

Grant-Mackie, J.A., 2013. Makoiamya cotterallae, a new genus and species of bivalve (Ceratomyidae) from the latest Triassic of New Zealand and New Caledonia. Zootaxa 3741, 327–348.

Hillhouse, J.W., and Gromme, C.S. (1984). Northward displacement and accretion of Wrangellia: New paleomagnetic evidence from Alaska. Journal of Geophysical Research, v. 89(B6), p. 4461–4477.

Jones, D.L., Silberling, N.J., and Hillhouse, J. (1977). Wrangellia - a Displaced Terrane in Northwestern North America. Canadian Journal of Earth Sciences, v. 14, p. 2565–2577.

Kozur, H., Mock, R., 1991. New Middle Carnian and Rhaetian conodonts from Hungary and the Alps, stratigraphic importance and tectonic implications for the Buda Mountains and adjacent areas. J. Geol. Bundesanst. 134, 271–297.

Maron, M., Onoue, T., Satolli, S., Soda, K., Sato, H., Muttoni, G., and Rigo, M.: Weathering trends in the Norian through geochemical and rock magnetic analyses from the Pignola-Abriola Section (Lagonegro Basin, Italy), Clim. Past, 20, 637-658.

Matsuda T, Isozaki Y. 1991. Well-documented travel history of Mesozoic pelagic chert in Japan: from remote ocean to subduction zone. *Tectonics* 10, 475-499.

McLennan, S.M., 2001. Relationships between the trace element composition of sedimentary rocks and upper continental crust. Geochem. Geophys. Geosyst. 2, 2000GC000109.

Monger, J.W.H. (1997). Plate tectonics and northern cordilleran geology: An unfinished revolution. Geoscience Canada, v. 24, p. 189–198.

Monger, J.W.H., and Ross, C.A. (1971). Distribution of Fusulinaceans in the Western Canadian Cordillera. Canadian Journal of Earth Sciences, v. 8, p. 259–278.

Muller, J.E. (1977). Evolution of the Pacific Margin, Vancouver Island, and Adjacent Regions. Can J Earth Sci, v. 14, p. 2062–2085.

Nesbitt H.W., Young G. M., 1982. Early Proterozoic Climates and Plate Motions Inferred from Major Element Chemistry of Lutites. Nature, 299, 715-717

Nixon, G.T., and Orr, A.J. (2007). Recent revisions to the Early Mesozoic stratigraphy of northern Vancouver Island (NTS 102I; 092L) and metallogenic implications, British Columbia. Geological Fieldwork 2006, BC Ministry of Energy, Mines and Petroleum Resources, p. 163–177.

Reggiani, L., Bertinelli, A., Ciarapica, G., Marcucci, M., Passeri, L., Ricci, C., Rigo, M., 2005. Triassic–Jurassic stratigraphy of the Madonna del Sirino succession (Lagonegro basin, Southern Apennines, Italy). Boll. Soc. Geol. Ital. 124, 281–291.

Rigo M., Campbell C. (2022). Correlation between the Warepan/Otapirian and the Norian/Rhaetian stage boundary: implications of a global negative δ^13^C_org_ perturbation. New Zealand Journal of Geology and Geophysics, 65(3), 397–406.

Rigo, M., Bertinelli, A., Concheri, G., Gattolin, G., Godfrey, L., Katz, M.E., Maron, M., Muttoni, G., Sprovieri, M., Stellin, F., Zaffani, M., 2016. The Pignola-Abriola section (southern Apennines, Italy): A new GSSP candidate for the base of the Rhaetian Stage. Lethaia 49 (3), 287–306. https://doi.org/10.1111/let.12145.

Rigo, M., De Zanche, V., Mietto, P., Preto, N., Roghi, G., 2005. Biostratigraphy of the Calcari con Selce formation. Boll. Soc. Geol. Ital. 124, 293–300.

Rigo, M., Mazza, M., Karádi, V., Nicora, A., 2018. New Upper Triassic conodont biozo- nation on of the Tethyan Realm. In: Tanner, L.H. (Ed.), The Late Triassic World: Earth in a Time of Transition. Topics in Geobiology 46. pp. 189–235.

Rigo, M., Onoue, T., Tanner, L.H., Lucas, S.G., Godfrey, L., Katz, M.E., Zaffani, M., Grice, K., Cesar, J., Yamashita, D., Maron, M., Tackett, L.S., Campbell, H., Tateo, F., Concheri, G., Agnini, C., Chiari, M. and Bertinelli, A., 2020. The Late Triassic Extinction at the Norian/Rhaetian boundary: Biotic evidence and geochemical signature. Earth-Science Reviews, 204: 103180.

Rigo, M., Preto, N., Franceschi, M., Guaiumi, C., 2012. Stratigraphy of the Carnian- Norian Calcari con Selce Formation in the Lagonegro Basin, Southern Apennines. Riv. Ital. Paleontol. Stratigr. 118, 143–154.

Sato H., Ishikawa A., Onoue T., Tomimatsu Y., Rigo M., (2021). Sedimentary record of Upper Triassic impact in the Lagonegro Basin, southern Italy: Insights from highly siderophile elements and Re-Os isotope stratigraphy across the Norian/Rhaetian boundary. Chemical Geology 586, 120506.

Scandone, P., 1967. Studi di geologia lucana: nota illustrativa della carta dei terreni della serie calcareo-silico-marnosa. Boll. Soc. Nat. Napoli 76, 1–175.

Stampfli, G.M., Vavassis, I., De Bono, A., Rosselet, F., Matti, B., Bellini, M., 2003. Remnants of the Paleotethys oceanic suture-zone in the western Tethys area. Boll. Soc. Geol. Ital. Spec. 2, 1–23.

Uno, K., Onoue, T., Hamada, K. & Hamami, S. 2015. Palaeomagnetism of Middle Triassic red bedded cherts from Southwest Japan: Equatorial palaeolatitude of primary magnetization and widespread secondary magnetization. *Geophys. J. Int.* 189, 1383–1398.

Zaninetti, L., Martini, R., Dumont, T., 1992. Triassic foraminifers from sites 761 and 764, Wombat Plateau, northwest Australia. Proc. Ocean Drill. Program Sci. Results 122, 427–436.
